# Evaluation and optimization of membrane feeding compared to direct feeding as an assay for infectivity

**DOI:** 10.1186/1475-2875-7-248

**Published:** 2008-12-02

**Authors:** Mouctar Diallo, Abdoulaye M Touré, Sekou F Traoré, Oumou Niaré, Lalla Kassambara, Awa Konaré, Mamadou Coulibaly, Magaran Bagayogo, John C Beier, Richard K Sakai, Yéya T Touré, Ogobara K Doumbo

**Affiliations:** 1 Malaria Research and Training Center (MRTC), University of Bamako, Bamako, Mali; 2 Department of Epidemiology of parasitic Diseases, currently at care Mali, Bamako, Mali; 3 World Health Organization, WHO/AFRO, Gabon; 4 Department of Tropical Medicine, Tulane University, New Orleans, LA, USA; 5 World Health Organization (WHO), Geneva, Switzerland; 6 Currently at Millennium Development Goal Center for West & Central Africa (MDG Center WCA), Bamako, Mali; 7 Currently at Miller School of Medicine, University of Miami, Florida, USA

## Abstract

**Background:**

Malaria parasite infectivity to mosquitoes has been measured in a variety of ways and setting, includind direct feeds of and/or membrane feeding blood collected from randomly selected or gametocytemic volunteers. *Anopheles gambiae s.l *is the main vector responsible of *Plasmodium falciparum *transmission in Bancoumana and represents about 90% of the laboratory findings, whereas *Plasmodium malariae *and *Plasmodium ovale *together represent only 10%.

**Materials and methods:**

Between August 1996 and December 1998, direct and membrane feeding methods were compared for the infectivity of children and adolescent gametocyte carriers to anopheline mosquitoes in the village of Bancoumana in Mali. Gametocyte carriers were recruited twice a month through a screening of members of 30 families using Giemsa-stained thick blood smears. F1 generation mosquitoes issued from individual female wild mosquitoes from Bancoumana were reared in a controlled insectary conditions and fed 5% sugar solution in the laboratory in Bamako, until the feeding day when they are starved 12 hours before the feeding experiment. These F1 generation mosquitoes were divided in two groups, one group fed directly on gametocyte carriers and the other fed using membrane feeding method.

**Results:**

Results from 372 *Plasmodium falciparum *gametocyte carriers showed that children aged 4–9 years were more infectious than adolescents (p = 0.039), especially during the rainy season. Data from 35 carriers showed that mosquitoes which were used for direct feeding were about 1.5 times more likely to feed (p < 0.001) and two times more likely to become infected, if they fed (p < 0.001), than were those which were used for membrane feeding. Overall, infectivity was about three-times higher for direct feeding than for membrane feeding (p < 0.001).

**Conclusion:**

Although intensity of infectivity was lower for membrane feeding, it could be a surrogate to direct feeding for evaluating transmission-blocking activity of candidate malaria vaccines. An optimization of the method for future trials would involve using about three-times more mosquitoes than would be used for direct feeding.

## Background

Studies of vector-parasite relationships under field conditions have helped to identify human reservoirs of infection[1,2] host-, parasite-, and vector-based determinants of infection[3]; the natural efficiency of sporogonic development in the mosquito vector *Anopheles gambiae*[4]; and naturally occurring transmission-blocking antibodies [1,5-7]. Most of these important studies have been performed with colony-reared mosquitoes or F1 progeny from field-collected mosquitoes which fed either directly on naturally-infected hosts or using a membrane-feeding apparatus through parafilm or 'baudruche' membranes on blood drawn from infected humans. Although mosquitoes can be infected using either feeding method, as determined by dissection of mosquito midguts for oocysts 7–8 days after feeding, the two methods have never been compared for efficiency of transmission. The degree to which the two methods reflect the natural transmission-blocking activity of host antibodies in the mosquito is unclear, because of potential differences in infectivity between *Plasmodium falciparum *gametocytes from human hosts and those from *in vitro *cultures[8], and because of potential differences in infectivity between F1 *An. gambiae *and laboratory strains of *Anopheles freeborni *or *Anopheles stephensi.*

Studies to date have examined human infectiousness to mosquitoes at three distinct levels [9]: (1) infectiousness of the individual according to gametocyte density, (2) the age group reservoir of infection within a site, and, most recently, (3) across populations of differing intensity.

Direct feeding has been the standard laboratory method for measuring transmission-blocking activity (the ability to block transmission from infected human hosts to mosquitoes) among candidate malaria vaccines. However, the direct feeding method could expose subjects to malaria infection if the mosquitoes are not reared under sterile conditions, raising ethical concerns – especially when children are involved in this type of study. Here are reported the results of a study conducted from 1996 to 1998 to evaluate the dynamics of transmission in a rural area of Mali using direct feeding and to then determine differences in infectivity rates between direct feeding and membrane feeding from the blood of young volunteers naturally-infected with *P. falciparum. *Results of this study, conducted as part of a larger epidemiological study of malaria in the area, could be used to inform study design for future vaccine trials using membrane feeding.

## Materials and methods

### Study design and recruitment of volunteers

**Figure 1:**
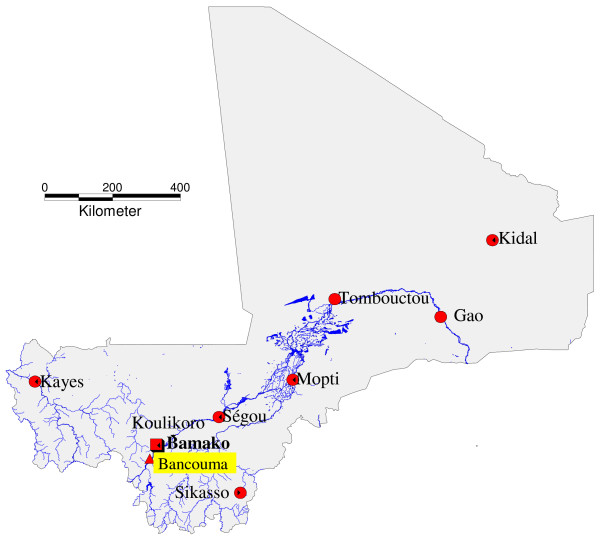


Children and adolescent volunteers were recruited to conduct a direct feeding experiment in Bancoumana to evaluate the dynamics of transmission between seasons and between age groups (4 to 9 years versus 10 to18 years). Comparison of infectivity between direct feeding and membrane feeding was also performed with a subset of these volunteers, in Bamako. Bancoumana, a village of approximately 10,000 inhabitants, is located 60 km from Bamako and 5 km from the Niger River in south-western Mali (12°20'N, 80°20'W). (Figure 1). The climate is soudanian with two seasons: a rainy season from June to October and a dry season from November to May. Transmission of malaria is intense during the rainy season and for a month or two thereafter. The major vectors are *An. gambiae s.s *(about 95.5%) and *Anopheles arabiensis *(about 4.5%) [10].

Twice a month during our study period, from August 1996 to December 1998, volunteers between four and 18 years of age were recruited from 30 families in Bancoumana. All volunteers were screened for *P. falciparum *by staining thick blood smears with Giemsa and counting gametocytes and parasites using a microscope. The 10 volunteers with the highest gametocyte levels were selected for the direct feeding experiment in Bancoumana. In 1998, two volunteers with the highest gametocyte levels at each time point were transported to Bamako for comparison of infectivity between direct feeding and membrane feeding.

Written informed consent was obtained from all volunteers or their guardians before enrollment in the study. Human subject's research conducted in our experiments was reviewed and approved by the Institutional Ethical Committee of the National School of Medicine and Pharmacy, Mali.

### Laboratory-reared *An. gambiae *mosquitoes

F1 progeny of wild-caught *An. gambiae *from Bancoumana were used for all experiments. The mosquitoes were reared in the insectary in Bamako, under controlled conditions of 28°C and 80% relative humidity. Because they were reared in a sterile laboratory environment, they remained uninfected before the experiment began. The mosquitoes were reared to the adult stage (three to six days of age) before being used in feeding experiments. After emerging, female mosquitoes were fed daily with a 5% sugar solution, up to 12 hr before blood feedings.

### Direct feeding method

For each volunteer, two paper cups containing 30 mosquitoes each were applied directly to the legs of volunteers, and the 60 mosquitoes were allowed to feed for 10–15 min. In Bancoumana, direct feeding was conducted in a dark room under natural temperature and humidity conditions, which fluxuated during the rainy and dry seasons. In Bamako, direct feeding was conducted in the insectary under constant temperature and humidity as described above. In both locations, we did not clean off the feeding site on the legs of the volunteers. Volunteers with local reactions such as pruritus were treated with an antihistaminic cream.

### Membrane feeding method

For the two volunteers selected at each time point for the comparison experiment, membrane feeding was performed using fresh venous blood. Briefly, 3 ml of blood was drawn from each volunteer and mixed with citrate phosphate dextrose. Then, two 1.5-ml aliquots were placed directly into a mosquito feeder within 5 min. Standard medium-sized membrane feeders were used, that used circulating water to maintain a temperature of 37°C. For each volunteer, two cups of 30 mosquitoes each were placed in the feeder, and the 60 mosquitoes were allowed to feed for 10–15 min. All membrane feedings were conducted in Bamako. After participating in the feeding experiments, volunteers were transported back to Bancoumana.

### Infectivity assessment among mosquitoes

After feeding on the blood of infected volunteers, non engorged mosquitoes (those with clear abdomens) were discarded, and engorged mosquitoes (those with red, distended abdomens) were retained under the insectary conditions in Bamako, described above, for 7–8 days to permit parasite maturation to the oocysts stage. Then mosquitoes were dissected and oocysts counted from the stomach using microscopy. By definition an infected mosquito is any mosquito with oocysts detected after dissection.

### Data management and statistical analysis

All data collected during screening were entered into Visual dBASE, SPSS 10.1 for Windows and Microsoft Excel. Parasite and gametocyte prevalence were then determined according to volunteers age. Mosquito data were analysed from information collected during feeding experiments. Effects of age and season on infectivity were determined from the direct feeding experiment using data from 1997 and 1998 only. Then, a comparison of infectivity was made between direct feeding and membrane feeding methods using data from 1998. For that, the comparison was done for the mean percentages of exposed mosquitoes that were engorged; the mean percentages of engorged mosquitoes that were infected; and the mean percentages of exposed mosquitoes that were infected. Mean percentages were calculated by averaging the percentages determined twice each month. All were calculated using regular means. Student t tests were used for all comparisons. A p value of 0.05 was considered significant.

## Results

### Malaria parameters

**Figure 2:**
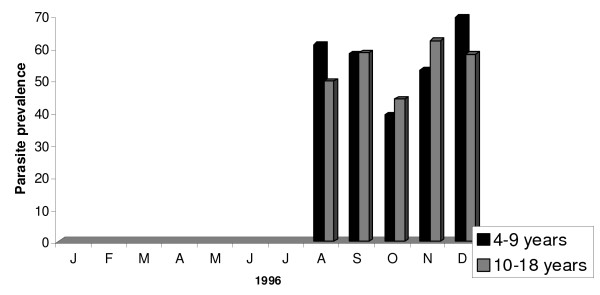


**Figure 3:**
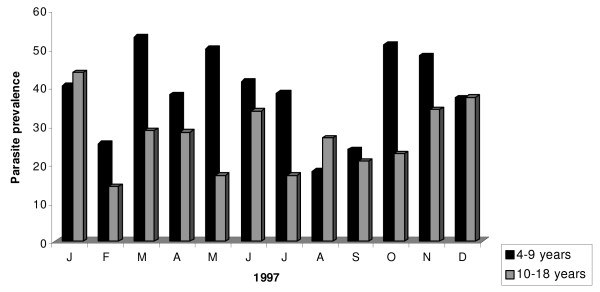


From August 1996 to December 1998, 4,826 children between four and 18 years were screened to determine parasite and gametocyte prevalence in Bancoumana. Figure 2 shows the monthly variation in parasite prevalence according to volunteer's age. In 1996, mean parasite prevalences were comparable between the two age groups (56.2% for the 4–9 year old versus 54.5% for the 10–18 year old; p = 0. 198). However, mean parasite prevalences were significantly higher in the younger age group for both 1997 (38.8%) and 1998 (27.1%) p = 0.004 for both years. Analogous results were obtained when we examined gametocyte prevalence according to age (Figure 3). The mean gametocyte prevalence was 14.5% in 1996, 11.8% in 1997, and 9.8% in 1998. No statistically significant difference was observed between these percentages. In contrast to mean parasite prevalence, mean gametocyte prevalence were significantly higher in the younger age group in all three years (p < 0.001).

### Direct feeding experiment

Among the 4,826 children and adolescents screened, 485 (10.0%) were identified as gametocyte carriers. Of these, 372 (76.7%) were included in the direct feeding experiment to determine an age or season effect on the infectivity of mosquitoes. While no significant differences were found in the mean percentages of mosquitoes that became engorged between the two age groups, no significant age effect on infectivity was find (Table 1). The mean percentage of engorged mosquitoes that became infected was significantly higher in the younger group than in the older group (38.3% versus 23.0%; p = 0.039).

**Table 1:** Age effect of gametocyte carriers on infectivity by direct feeding in Bancoumana, Mali

**Age group (y)**	**N**	**Mean % engorged/all mosquitoes**	**Mean % infected/engorged mosquitoes**	**Mean % infected/all mosquitoes**
4–9	236	55.8	38.3	19.7
10–18	87	55.4	23.0	12.1
		p = 0.525	p = 0.039	p < 0.001

Total	323	55.7	34.2	17.7

When, infectivity according to season was compared, no statistically significant difference in the mean percentages of mosquitoes that became stuffed in the dry season versus the rainy season was observed. However, a higher mean percentage of engorged mosquitoes did become infected during the rainy season than during the dry season, but this difference also was not significant (Table 2).

**Table 2:** Seasonal effect on infectivity of mosquitoes by direct feeding in Bancoumana, Mali (1997–1998)

Season	N	Mean % engorged/all mosquitoes	Mean % infected/engorged mosquitoes	Mean % infected/all mosquitoes
**Dry Season**	159	56.1	29.0	16.7
**Rainy Season**	164	55.3	39.2	18.6
		**P = 0.738**	**p = 0.120**	**p = 0.402**

Total	322	55.7	34.2	17.7

### Direct feeding versus membrane feeding experiment

Of the 372 volunteers in the direct feeding experiment, 35 (9.4%) were included in the comparison of infectivity between direct feeding and membrane feeding. Results showed that mosquitoes using the direct feeding method were significantly more likely to become engorged than were those using the membrane feeding method (mean percentages, 76.0% versus 53.5%). In addition, those that became engorged via direct feeding were significantly more likely to become infected than were those engorged via membrane feeding (mean percentages, 28.4% versus 15.0%). Of all mosquitoes in the experiment, those used in the direct feeding were almost three times more likely to become infected than were those used in the membrane feeding (Table 3). Applying the Student t test, there was no statistically significant difference between the percentage of gametocyte carriers, who were infectious to mosquitoes using the direct feeding technique [(94.2%) and the membrane feeding technique (85.7%); p = 0.5].

**Table 3:** Comparison of infectivity between direct and membrane feeding in Bancoumana, Mali

Method	Mean % engorged/all mosquitoes	Mean % infected/engorged mosquitoes	Mean % infected/all mosquitoes
**Direct feeding**	76.0	28.4	20.6
	**P < 0.001**	**P < 0. 001**	**P < 0.001**
			
**Membrane Feeding**	53.5	15.0	7.6

## Discussion

Because of study implementation difficulties, data from 1996 were not included in this analysis. In the first component of the study the direct feeding was used to evaluate the dynamics of transmission among children aged 4–18 years in rural Mali. Results showed that among 372 gametocyte carriers analyzed, those aged 4–9 years were highly infectious to mosquitoes when compared with those aged 10–18 years, particularly during the rainy season.

In the 1950s, Muirhead-Thompson [11,12] was the first to publish an attempt to determine the proportion of individuals in a malaria-endemic area, who are infectious to mosquitoes. He found that 10–11% of all individuals in West Africa were infectious to mosquitoes by direct feeding. Because the infectivity rates in children were significantly higher than those in adults (34% of children aged 2–4 years and 37% of children aged 5–9 years infected mosquitoes in that study), he proposed that children represented the major infectious reservoir of malaria parasites. Also, Bousema *et al *[13] found that the estimated mean duration of gametocytaemia was 9.4 days (range 2.5 – 23.5) for children below five years of age, 7.8 days (range 2.5 – 23.5) for children aged five to nine years and 4.1 days (range 2.5 – 16.5) for children aged ten years and above. A subsequent study by Githeko *et al *[2], in a holoendemic area of western Kenya, confirmed and extended the previous results. In that study, 72% of the infectious reservoir was in children younger than 10 years. Moreover, Boudin *et al *[14] found that neither the proportions of reducers nor the medians of transmission blocking immunity (TBI), intensity, or TBI/TI (transmission inhibition) were significantly different between age groups. And thus, age was not predictive for any of the TBI indicators.

The second component of the study, which included 35 gametocyte carriers, compared infectivity rates via direct versus membrane feeding of mosquitoes. A statistically significant difference was observed between the mean percentage of mosquitoes that fed by direct feeding (76.0%) and the mean percentage of mosquitoes that fed by membrane feeding (53.5%), suggesting that mosquitoes preferred to use direct feeding. These results are comparable to those found by Graves *et al *[5,6] in 1988. That research also found that for both techniques, 82.82% of all carriers were infectious to mosquitoes. Again, this is similar to our findings that 94.2% and 85.7% of children were infectious by direct and membrane feeding, respectively (with no significant difference between the two values).

However, a statistically significant difference was found between the mean of percentages of engorged mosquitoes infected by direct feeding (28.4%) versus membrane feeding (15.0%). In Cameroon, Mulder *et al *[1] observed that a mean of 18.9% of engorged mosquitoes were infected by membrane feeding. Overall, this study shows that, membrane feeding is about half as effective as direct feeding for infecting mosquitoes. And about three times more mosquitoes in the direct feeding group than in the membrane feeding group actually became infected during feeding; this finding can be explained, at least in part, by the fact that mosquitoes preferred direct feeding.

## Conclusion

Based on these results, the conclusion is that the membrane feeding method could be used as an alternative to the direct feeding method in biological studies or field trials of transmission-blocking activity among candidate malaria vaccines. Although the intensity of infection is considerably higher when the direct feeding method is used, the difference in infectivity between the two methods could be compensated for by including more mosquitoes in trials using membrane feeding. Using this study as an example, if three-times more mosquitoes had been exposed to membrane feeding, it is expected that the number of mosquitoes infected would have been similar for both methods. The lower mean percentage of mosquitoes infected by membrane feeding may result from damage during blood drawing and handling, or from loss of infectivity while the parasites are in the membrane feeders. Nevertheless, the membrane feeding technique is easily applicable, and its use would resolve the ethical concerns surrounding the direct feeding technique, especially among children.

## Competing interests

The authors declare that they have no competing interests.

## Authors' contributions

MD conducted parsitotolgic aspects of the study in the field and in laboratory, and initiated the draft zero; AMT conducted entomologic aspects of the study in the field and in the laboratory, and contributed to writing all of the drafts; SFT supervised mosquitoes feeding in the laboratory; ON supervised mosquitoes breeding in the laboratory (insectary) and dissection; LK conducted the blood sample collection from volunteers; AK conducted the blood sample collection from volunteers; MC contributed to mosquitoes dissection; MB contributed to mosquitoes dissection, entomology studies, and direct feeding; OkD, SK, JCB, and YTT reviewed the final version

## Financial support

This investigation received financial support from Tropical Medicine and Research Center (TMRC) project of Mali-Tulane, TMRC AI 95002.
